# *Alternaria radicina*; unveiling the cause, spread, and molecular basis of a novel coriander leaf blight disease in Egypt

**DOI:** 10.1016/j.heliyon.2024.e41081

**Published:** 2024-12-10

**Authors:** Khalid M. Ghoneem, Ehsan M. Rashad, Abdulaziz A. Al-Askar, Yosra A. Helmy, Seham M.A. El-Gamal, Shafik D. Ibrahim, WesamEldin I.A. Saber

**Affiliations:** a Department of Seed Pathology Research, Plant Pathology Research Institute, Agricultural Research Center, (ARC), Giza, 12619, Egypt; b Department of Botany and Microbiology, Faculty of Science, King Saud University, Riyadh, 11451, Saudi Arabia; c Department of Veterinary Science, Martin-Gatton College of Agriculture, Food, and Environment, University of Kentucky, Lexington, KY, 40546, USA; d Department of Medicinal and Aromatic Plants Research, Horticulture Research Institute, Agricultural Research Center (ARC), Giza, 12619, Egypt; e Department of Genome Mapping, Agricultural Genetic Engineering Research Institute (AGERI), Agricultural Research Center (ARC), Giza, Egypt; f Microbial Activity Unit, Department of Microbiology, Soils, Water and Environment Research Institute, Agricultural Research Center (ARC), Giza, 12619, Egypt

**Keywords:** *Alternaria* specific gene, Enzymes, Molecular identification, Seed-borne fungi

## Abstract

*Coriandrum sativum* L. faced a new and previously undocumented leaf blight disease for the first time. This disease manifests initially as small, circular, or irregular brown spots on older leaves, which gradually expand and merge into dark brownish blotches over time. This disease's impact is detrimental to plant health and seed quality. Through comprehensive morphological characteristics, and molecular identification (GenBank OL823169), the pathogen was conclusively identified as *Alternaria radicina*. Further confirmation was obtained by analyzing target sequences for the *Alt-a-1* gene (GenBank OR492259). Koch's postulates were strictly adhered to, leading to the successful re-isolation of *A. radicina* from artificially infected 8-week-old coriander plants (local variety; Balady), providing unequivocal evidence that the fungus is responsible for leaf blight disease, marking the first such confirmation worldwide. The significant activity of fungal enzymes may be associated with pathogenicity. The seed-health test supported the hypothesis that seeds play a central role in the transmission of *A. radicina*, as it was detected in 40 % of seed lots, alongside other common pathogenic and saprophytic genera. This study underscores the urgency of implementing seed treatments to curtail the pathogen's spread. The emergence of coriander leaf blight disease documented here (Egypt; 30° 57′ 25″ N and 31° 35 ′ 54″E) for the first time, necessitates heightened awareness and proactive measures to protect coriander plants all over the world.

## Highlights

• New coriander disease caused by *Alternaria. radicina*.
• Koch's postulates confirmed the pathogenicity.
• Coriander seeds spread coriander blight.
• Urgent seed treatment.

## 1 Introduction

*Coriandrum sativum* L., (family; *Apiaceae*), is an aromatic herbaceous annual plant, mainly cultivated in winter for its fruits (seeds) and leaves, as well. It is used for medicinal and culinary purposes, representing an indispensable spice in different cuisines [1,2]. The plant is indigenous to the Mediterranean region and is commonly grown in North Africa, Central Europe, Russia, and Asia [2]. Many phytochemical studies have shown that coriander is rich in vitamin B12, folate, vitamin C, vitamin A, linalool, and phenolics. Its fixed oil is rich in tocols, bioactive phytochemicals, and sterols [3]. Different parts of the coriander plant are used as flavoring agents and as a remedy for healing several gastrointestinal complaints, e.g. anorexia, flatulence, dyspepsia, pain, vomiting, and diarrhea [2,4]. As a medicinal plant, various pharmacological influences have also been ascribed i.e., antimicrobial, antioxidant, antidiabetic, anti-epileptic, anxiolytic, antidepressant, anti-inflammatory, antimutagenic, antidyslipidemic, neuroprotective, diuretic, antihypertensive, and detoxifying potential [1,4]. Coriander essential oil (0.03–2.6 %) has a high content linalool and is thus used in body care products e.g., cosmetics, and perfumes [4,5]. Because of the suitable environment, Egypt is a major grower of medicinal, and aromatic plants in the Middle East [6], with an area of 9500 acres, representing 12.6 % of the total planted area [7]. Egyptian coriander exports have surged by a substantial 119.2 %, increasing from 407.6 tons in 2017 to 893.6 tons in 2022 [8].

Unfortunately, several phytopathogenic fungi attack coriander plants causing leaf spot diseases worldwide like, *Cladosporium tenuissimum*, *Alternaria alternata, Alternaria dauci*, *Phoma multirostrata*, *Colletotrichum gloeosporioides, C. capsica, Cercospora corianderi*, and *Ulocladium oblongoobovoideum* [[9], [10], [11], [12]].

The genus *Alternaria* includes many species with various pathogenic abilities [13], and the majority are transmitted by seeds [14], initiating the damping-off of numerous *Apiaceae* seedlings [13,15,16]. *Alternaria radicina* is known primarily as a seed-borne pathogen of most *Apiaceae* plants (carrot, parsnip, celery and celeriac, caraway, dill, parsley, and fennel), causing foliar blight and stalk/root rot disease [13,[17], [18], [19], [20]]. It is also reported to produce harmful mycotoxins [21,22]. In Egypt, the pathogen was reported on anise seeds [23]. Greenhouse assays revealed that the pathogen caused severe leaf spot disease on three-month-old anise plants, with disease incidence and severity reaching 65.5 % and 2.73, respectively, in the first season, and 82.55 % and 2.85 in the second [24]. This pathogen is widespread, affecting carrots at all growth stages and during storage, with up to 47 % of seedlings and 88 % of mature plants infected [15,25]. Under optimal conditions, disease spread can reach 70–80 %, reducing yield by 35–50 %, and stored seeds can be completely lost by spring [25]. Beyond carrots, the pathogen infects other Apiaceae members, causing significant losses [26].

Fungal pathogens have lytic activity on plant tissue, recognized as cell-wall destroying enzymes – that aid the invasion of fungi into the hard-to-degrade tissues of plants [[27], [28], [29]]. The enzymatic profile of a microorganism differs greatly from one to another based on the genetic structure, the ecological conditions, and the host, consequently, enzymes are considered one of the key features of microbial pathogenicity [28,29].

Coriander is a crucial economic crop in Egypt, contributing significantly to the country's agricultural sector. However, recent outbreaks of a new leaf blight disease have led to significant yield losses, posing a serious threat to coriander production. The initial symptoms of the foliar blight disease appear consistent with those caused by *Alternaria* fungi, suggesting a potential association with this genus. To date, the causative agent of this disease remains unidentified, hindering effective disease management strategies.

This study aims to isolate and characterize the pathogen responsible for the coriander leaf blight that recently emerged in Egypt. Morphological and molecular techniques, including the analysis of the ITS region and the major allergen gene (*Alt-a-1*) of *Alternaria* spp., were employed for pathogen identification. Additionally, seed health tests were performed to investigate the potential role of seeds in pathogen transmission. By unveiling the identity of the pathogen and its mode of spread, this research provides essential information for developing sustainable control measures to mitigate the impact of coriander leaf blight on Egyptian agriculture.

## 2 Materials and methods

### 2.1 Collection and isolation of the pathogen

The inspection trial, for isolation of the pathogens, was conducted on sixteen infected plants having symptoms of leaf blight and gathered from four different growing areas belonging to two governorates in middle Egypt from the beginning of January till the middle of April 2021. The collected infected plants showed a severe foliar blight with an incidence of 40–70 % and a severity of 20–40 %, (n = 150). Samples from each location were collected randomly in a zigzag pattern. The disease symptoms were recorded for the first time in Tagelzz Research Station, ARC, Dakahlia governorate, Egypt (30° 57′ 25″ N and 31° 35′ 54″E), then spread to other plantation areas, including two locations in El Baramon research station, HRI, ARC, Dakahlia governorate (31° 08′ 11.3″ N and 31° 28′ 19.6″) and Alshaeir Island Farm, El-Qanater El-kayreya, Qalyubia governorate (30° 91′ 33″ N and 31° 13 ′ 69″E). The collected plants were placed in numbered paper bags, stored in a cool box for transportation, and then kept at 4 °C until testing.

Infected coriander plant parts (leaf, stem, and umbel) were splashed with sterilized water and cut into fragments of approximately 0.5–1.0 cm, before surface sterilization by immersion in 2 % NaOCl (3 min), followed by 2–3 times rinsing in sterile distilled water. Five fragments were placed on PDA (potato dextrose agar) plates containing 0.3 mg/L streptomycin sulfate, and 0.1 mg/L L-chloramphenicol, as an antibacterial agent, and kept at 25 ± 2 °C, under cool white fluorescence light with 12/12 h, light/dark cycles for 5 days. To obtain pure cultures, the single spore and/or hyphal tip protocol were applied. The isolated *Alternaria* spp. were characterized based on macroscopic and microscopic characteristics, comprising size, number and color of transverse and longitudinal walls, presence of surface ornamentation, and tip, length, and size of conidiophore, as well as the presence of secondary conidiophore [30,31]. The purified fungi were grown on PCA (potato carrot agar) slants and preserved at 4 °C after growth.

### 2.2 Cultural and morphological characterization

For the characterization of the recovered fungi, monoconidial sub-cultures of each isolate were established on agar plates. Initially, the fungal isolates were grown on nutrient-deficient agar (SNA) [32], vegetable juice agar (V8) [33], and acidified potato dextrose agar (APDA) [34] at 22 °C under dark conditions. The grown cultures were investigated during the period from 10 to 21 days. The texture, color, and borders of the colonies were visually evaluated. After an eight-day culture period, the colonies' growth was measured. The conidiophores and apical conidia were differentiated and examined with a binocular (Olympus corporation SZ2-ILST, Japan) for i) branching status, and ii) flexible or erected. After 10-day growth on V8, the conidia generation intensity, as well as the width, and length of the conidia were measured by the light microscope (Olympus CX41 (Olympus Corporation, Japan), applying the program; AxioVision MTB2004 (Carl Zeiss AG, Germany). Conidia were classified into four types based on whether they had two, three, four, or five transepta. For each isolate, around 50 conidia from each transeptal category were counted. The yellow pigmentation (because of the toxin; radicinin which may turn into crystals), and microsclerotia were visually investigated in Petri dishes after 21 days. For each isolate and each medium, 6 replicates were used.

### 2.3 Pathogenicity test

The inoculum of 3 isolates (Ar77, Ar10, and Ar8) was obtained from 10 days-old conidia grown on PDA. The three selected isolates were characterized by strong growth, sporulation, and the release of yellow-colored pigments on acidified APDA media with no crystal production (type B isolate).

**Fig. 1:**
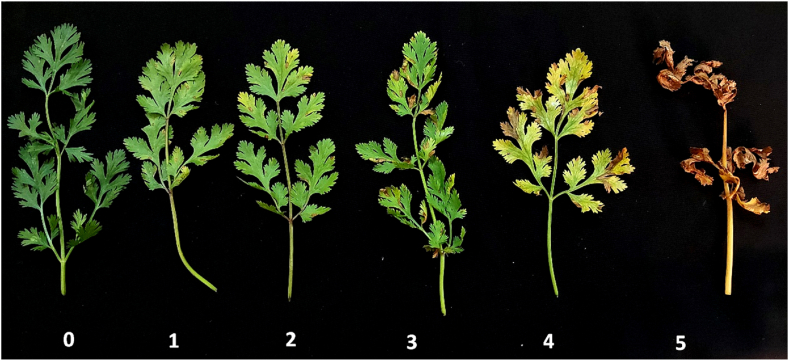
Coriander leaves infected with *Alternaria radicina* at various degrees, showed a severity index of 0 = healthy, 1 = 1–20 % infection, 2 ≥ 20–40 % infection, 3 ≥ 40–60 % infection, 4 ≥ 60–80 % infection, and 5 ≥ 80–100 % infection (100 % infection = complete death).

Under greenhouse conditions (25 ± 2 °C), the conidial suspension (10^5^ conidia/ml) was splashed onto 8-weeks-old healthy coriander plants (local variety; Balady) grown on sterilized soil. A control group of 10 plants was splashed with sterilized water. Plants of the two treatments were enclosed in plastic bags for 24 h to sustain high humidity. Observation lasted for 14 days under the greenhouse conditions (22 ± 3 °C). The severity of *Alternaria* leaf spots by each different three isolates was scored for the disease incidence (DI), and the disease severity index (DS) of the infected leaves. In respect to DI, a leaf is infected if one or more lesions are observed. An index key proposed scale of 0–5 level described by authors (Fig. 1) for *Alternaria* leaf blight on coriander was adapted as follows; 0 = healthy, 1 = 1–20 % infection, 2 ≥ 20–40 % infection, 3 ≥ 40–60 % infection, 4 ≥ 60–80 % infection and 5 ≥ 80–100 % infection (100 % infection = complete death). The authors proposed this scale due to the wide range of infection levels in coriander plants and the lack of similar scales for infection by the casual *Alternaria* pathogens among other plants in the Apiaceae family. The DS average for each plant was calculated from the sum of the DS of each leaf. To rate the damaged leaf areas, xerographic copies were made for the indexed infected leaves. Estimation of DS was performed by comparing the copies to the assigned damage scale, and then the averages of the DS were estimated. Each isolate was evaluated in three pots, constituting a replication. The experiment was repeated thrice with ten replications of each *Alternaria* isolate.

### 2.4 Molecular confirmation of pathogen

#### 2.4.1 Analysis of rDNA

An attempt was made to identify the most virulent isolate (Ar77) through the molecular approach of rDNA sequencing using fungal universal primers. Molecular identification was achieved by analyzing the internal transcribed spacer (ITS) ribosomal RNA. The genomic DNA extracted from the most severe isolate (*A. radicina* Ar77) served as a template for amplifying the ITS rRNA gene. This amplification was carried out using the two primers of ITS1 (5′-TCTGTAGGTGAACCTGCGG-3′) and ITS4 (5′-TCCTCCGCTTATTGATATGC-3′). The polymerase chain reaction (PCR) mixture consisted of 25 μl Master Mix (Sigma), 2 μl of each primer (10 pmol/μl), and 3 μl of template DNA (10 ng/μl), with the final reaction volume adjusted to 50 μl using ultra-pure water.

The PCR amplification, conducted with a PerkinElmer/GeneAmp® PCR System 9700 (PE Applied Biosystems), initiated with a primary denaturation process (5 min at 94 °C), then 40 denaturation sets (94 °C for 40 s), annealing (93 °C for 40 s), and elongation (72 °C for 50 s). Finally, an extension of the primer segment at 7 min at 72 °C was performed.

The amplified PCR products resulted using electrophoresis in a 1.5 % agarose gel stained with (0.5 μg/mL) ethidium bromide in 1× TBE buffer at 95 V for 1 h. PCR products were envisioned and photographed using UV light and a Gel Documentation System (BIO-RAD 2000).

The obtained PCR product sequence was aligned with the corresponding ITS rRNA sequences of the representative strain type in the genus, using the BLAST tool on GenBank. The evolutionary relationship was established using the Neighbor-Joining method [35], and the related taxa were clustered in the bootstrap test [36]. The uncertain positions of each pair of sequences were eliminated (pairwise deletion), and sixteen nucleotide sequences were compared using the Jukes-Cantor method [37]. MEGA11 software was used [38].

#### 2.4.2 *Alt-a-1* gene detection

The sequencing of the *Alt-a-1* specific gene of *A. radicina* Ar77 was inspected. Amplification of the PCR was performed in a mix of 25 μl Master Mix, 2 μl primer Alt-F (5′ ATGCAGTTCACCACCATCGC 3′), 2 μl primer Alt-R (5′ ACGAGGGTGAYGTAGGCGTC 3′) (10 pmol of each primer), 3 μl template DNA (10 ng), and 15 μl dH_2_O [39].

The PCR conditions, conducted with a PerkinElmer/GeneAmp® PCR System 9700 (PE Applied Biosystems), initiated with a primary denaturation process (3 min at 94 °C), then 40 denaturation sets (94 °C for 30 s), annealing (52 °C for 30 s), and elongation (72 °C for 50 s). Finally, an extension of the primer segment at 7 min at 72 °C was performed [39].

The amplified PCR products resulted using electrophoresis in a 1.5 % agarose gel stained with (0.5 μg/mL) ethidium bromide in 1× TBE buffer at 95 V for 1 h. PCR products were envisioned and photographed using UV light and a Gel Documentation System (BIO-RAD 2000).

The sequence of *Alt-a-1* was aligned with similar sequences in the GenBank. The multiple alignments with the top homologous hits were established using the neighbor-joining method with the MEGA 11 program. The alignment result was utilized to create a phylogenetic tree using BLAST pairwise alignments.

### 2.5 Enzymatic profile of *A. radicina* Ar77

#### 2.5.1 Culturing conditions

The hydrolytic enzymes produced by the pathogenic fungus were tested by cultivation under solid-state fermentation according to Saber et al. [40] with some modifications. This technique was used to mimic the natural conditions for fungal growth and infection. The desiccated coriander biomass was pulverized and employed, as a substrate, in the fermentation medium. Specifically, 1.0 g dried plant material was combined with 5 mL of tap water at pH 6.5 in 250 mL Erlenmeyer flasks and autoclaved at 121 °C for 15 min. For medium inoculation, two 0.5-mm discs of 5-day-old culture (grown on PDA plates) were used to inoculate the medium. A set of control flasks were applied using the same fermentation medium without fungal inoculation. All flasks (inoculated or not) were incubated (30 °C, 10 days). Sterilized water was added to the medium when needed to keep humidity (2.0 mL/flask, on the 5th day). Then, each flask was mixed with Tween 80 (0.01 %) to a total volume of 10 mL and shaken for 30 min at 150 rpm. The residual biomass and fungal growth were separated by filtration (Whatman No1), then centrifugation (20 min at 5000 rpm). The resulting fungal filtrate was assayed for enzymatic activity.

#### 2.5.2 Detection of lytic enzymes

Lytic enzyme activities (cellulase, xylanase, polygalacturonase, α-amylase, and protease) were determined according to standard protocols [[40], [41], [42], [43]]. Briefly, cellulase, xylanase, polygalacturonase, and α-amylase activities were assayed by measuring the release of reducing sugars from their respective substrates (microcrystalline cellulose, xylan, pectin, and starch) using the dinitrosalicylic acid method [44]. Protease action was assayed by measuring the increase in absorbance at 280 nm due to the release of amino acids from casein [45]. Detailed assay conditions, including substrate concentrations, incubation times, and temperatures, are provided in the Supplementary Materials (Section 1). Enzyme activities were expressed as units (U) per gram biomass/min.

### 2.6 Detection of *Alternaria radicina* through seed health testing

To clarify the role of seeds in transmitting a causal pathogen, 15 coriander samples from the coriander-growing governorates in Egypt including Al-Dakahlia, Al-Giza, Al-Menia, Assiute, and Qena. Sampling was performed in a 50 × 50 m area in a zigzag shape. The mature coriander umbels were placed in paper bags and stored at 4 °C. Coriander seeds were extracted from umbels and dried at 25 ± 2 °C for a few days on a porcelain plate. For each sample, 400 seeds from each harvested area were randomly collected and subjected to testing for the presence of the pathogen.

The seed health testing was performed using the standard moist blotter (SMB) method approved by the International Seed Testing Association [46]. Ten coriander seeds were distributed in a 9-cm-diameter sterile Petri dish, holding 3 layers of wet blotting paper, then incubated (20 ± 2 °C, 7 days) under white, fluorescent light with a 12 h turning cycle of light/dark. Seven days after incubation, the recovered fungi were recognized based on the habit features with a light-supported stereoscopic microscope (Olympus corporation SZ2-ILST, Japan).

The obtained pure fungi were transferred to plates of PDA. Other pure fungal cultures were transported to slants of PCA. The frequency (Equation (1)) and the average of the sample infections (Equation (2)) were estimated as follows:

Equation (1) $$\text{Frequency}\;\left(F\;\%\right)=\frac{\text{No}.\;\text{of}\;\text{infected}\;\text{samples}}{\text{Total}\;\text{No}.\;\text{of}\;\text{tested}\;\text{samples}}\times 100$$

Equation (2) $$\text{Mean}\;\text{of}\;\text{sample}\;\text{infection}\;\left(I\;\%\right)=\frac{\sum \text{fungus}\;\text{incidence}\;\text{in}\;\text{all}\;\text{examined}\;\text{samples}}{\text{Total}\;\text{No}.\;\text{of}\;\text{examined}\;\text{samples}}$$

Coriander seeds were subjected to SMB to assess seed health. As the most widely used seed health assay, SMB detects seed-borne fungi through incubation and subsequent observation. Emerging fungal colonies were transferred to PDA for pure culture isolation. Fungi were identified based on morphological characteristics observed under a stereomicroscope and compound microscope, including colony appearance on PDA and fungal structures on the seed surface. For precise species identification, particular attention was given to the presence of fruiting bodies and/or sporulation on infected seeds or in well-developed pure cultures. This approach enabled the detection of common seed-borne fungi such as *Alternaria, Bipolaris, Curvularia*, and *Fusarium*. Fungal identification adhered to standard mycological protocols [30,31,[47], [48], [49], [50], [51]].

### 2.7 Statistical analysis

Each trial was performed a minimum of three times, and the resulting means were calculated. To assess the frequency and incidence of the recovered seed-borne mycoflora of coriander, a heatmap was generated using the Minitab software package (Minitab Inc., ver. 22, USA).

## 3 Results and discussion

### 3.1 Description of the foliar blight symptoms

**Fig. 2:**
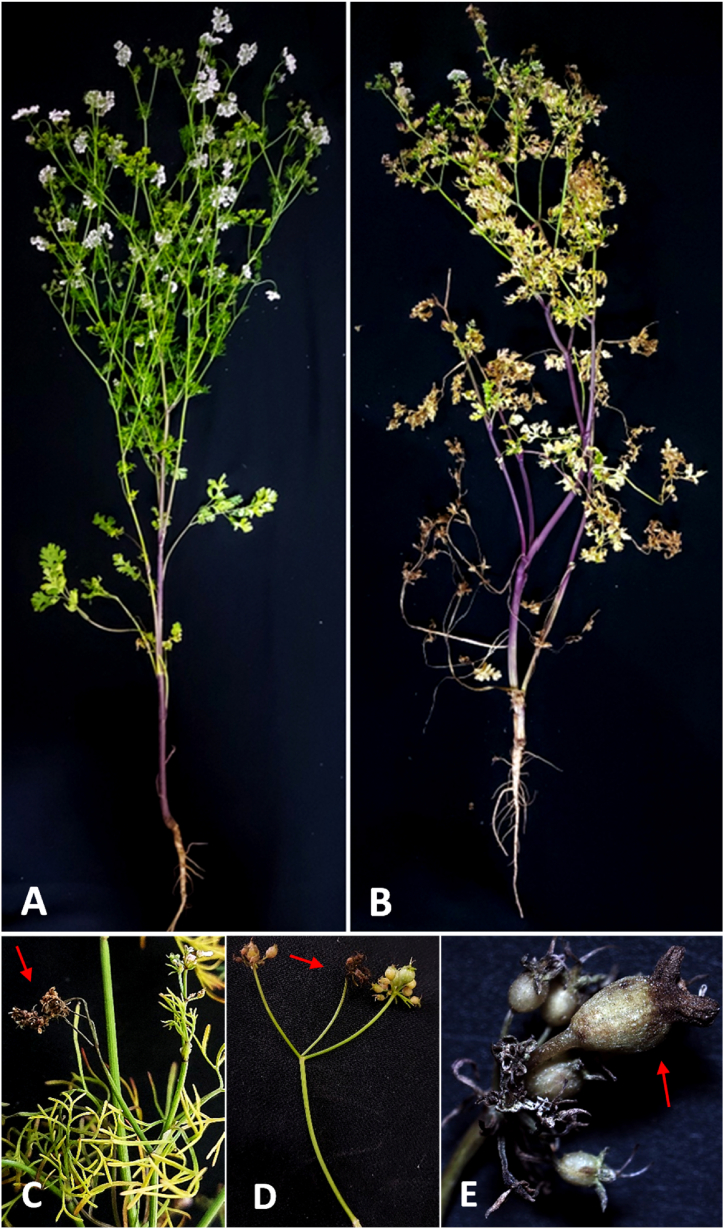
Natural infection symptoms caused by *Alternaria radicina* on coriander plants, showing A), healthy plant, B) infected plant, C) Close-up on stem necrosis, leaf, and umbellet inflorescence blight, D) and E) blighted umbellets (arrows).

**Fig. 3:**
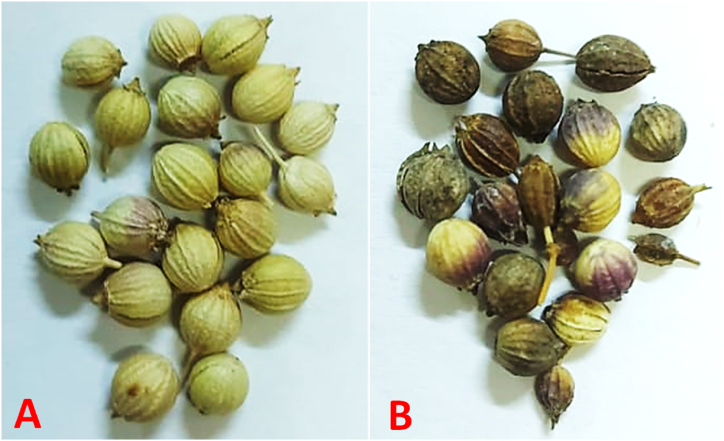
Natural infection symptoms caused by *Alternaria radicina* on coriander seeds, showing A), healthy seeds, and B) infected seeds appearing symptoms of discoloration, smaller size, and shriveling.

A severe foliar blight with an incidence of 40–70 % and severity of 20–40 % (n = 150) was observed, from the beginning of January till the middle of April 2021, to outbreak in coriander plantation areas of Dakahlia governorate (El Baramon, and Tagelzz research stations), and Qalyubia governorate (El-Qanater El-kayreya), Egypt. Symptoms emerged as tiny round or irregular brown spots on the oldest leaves at the plant base towards the top. As time progressed, they developed into small lesions with necrotic brown centers, then enlarged gradually and coalesced into brownish blotches. Damaged leaves became yellow, turned brown, and then died. The blight symptoms appeared also on the branches, stems, and umbels' necks as brown blotches, leading to death. In addition, some umbels showed a partial infection in which some florets died starting from the floret's neck as brown necrosis (Fig. 2A–E). In comparison to the healthy seeds, the infected seeds showed discoloration, smaller size, and shriveling (Fig. 3A and B).

### 3.2 Isolating and identifying *A. radicina*

**Fig. 4:**
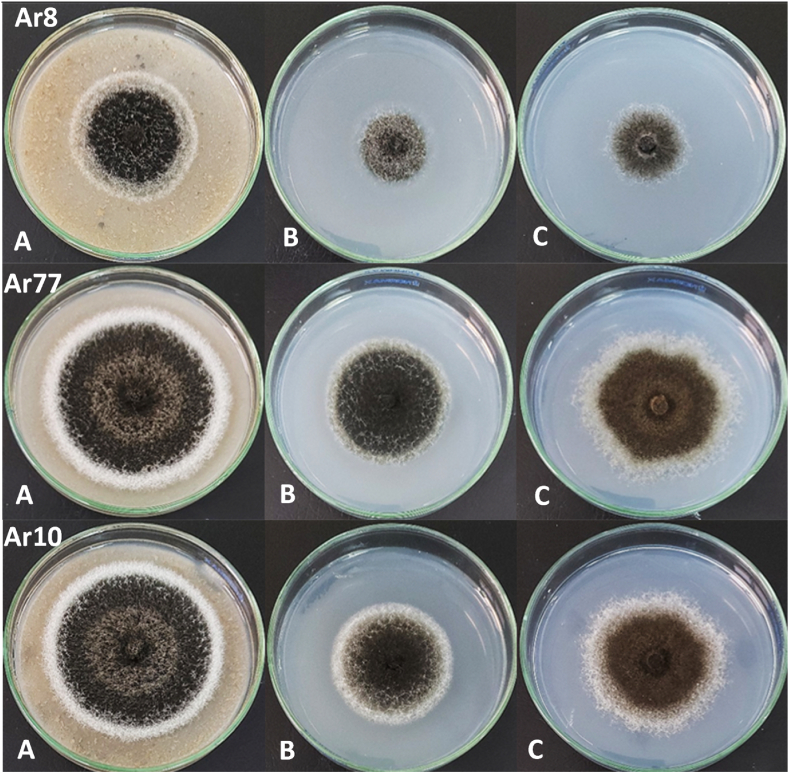
Colony morphology of *A. radicina* isolates Ar8, Ar77, and Ar10 on growth media after 10 days of incubation, (A) vegetable juice agar (V8), (B) acidified potato dextrose agar, and (C) synthetic nutrient deficient agar (SNA).

The isolation trial of the causative pathogen from coriander plants revealed three pathogenic isolates. Based on the morphological and cultural characteristics investigated on different media, the three selected pathogens were identified as *A. radicina* (Ar77, Ar10, and Ar8). After 10 days on V8 medium at 25 ± 2 °C (Fig. 4A–C), the isolates formed effuse and dark, blackish brown to black colony (54.7–61.3 mm in diameter). On acidified APDA, the fungus displayed moderate growth with an even margin, accompanied by the release of a slightly yellow-colored pigment [52]. No dendritic crystals were observed on the underside of the Petri dish and the growth never occupied the entire Petri dish after 21 days of incubation [53]. The fungus forms unique black colonies with irregular margins on synthetic SNA medium [32].

Three discrete types (a, b, and c) of *A. radicina* (*Stemphylium radicinum* (Meier. Drechs. and E.D. Eddy) have been identified based on colony morphology, spore characteristics, and host range [54]. Types “a” and “b” are highly pathogenic to carrots, while type “c” primarily affects celery and parsley. Type “a” isolates form small, irregular colonies with tree-like crystals on malt agar, whereas type “b” colonies are larger with smooth margins and lack crystals. The crystals produced by type “a” are composed of radicinin, a phytotoxic and antifungal compound. Most carrot isolates (>98 %) belong to type “a” and produce a yellow pigment on PDA [53]. While *A. radicina* and *A. carotiincultae* are similar, they can be differentiated by their growth rates and crystal production on acidified PDA. *A. radicina* grows slower, produces crystals, and has higher radicinin levels, making these characteristics useful for identification [55].

**Fig. 5:**
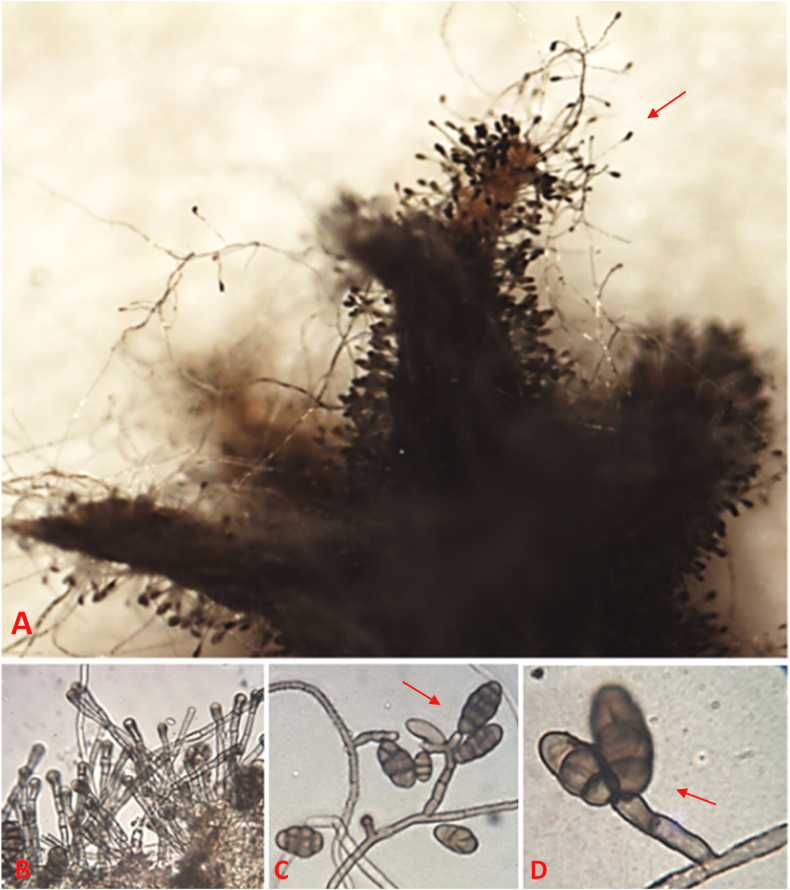
Micrograph stereoscopic of *Alternaria radicina* on coriander illustrates a seed surface entirely covered by conidiophores carrying conidia (arrow) at 64× magnification (A). Microscopic photographs reveal conidiophores emerging from hyphae at 400× (B), along with typical erect conidiophores bearing conidia (arrows) at 400× (C) and 1000× (D).

The conidiophores are erected and far separate from each other. The conidiophores generate sole apical conidia or, infrequently, clumps of single conidia. The apical conidia of the Ar77 strain were frequently branched on V8 (Fig. 5A–D, and Table 1). Based on variances in colony morphology and, to some extent, the morphology of spore, and host range, type “b” isolate was characterized by a large colony with equal margins and commonly did not create crystals [53,54]. Comparable results were found by Kathe et al. [56], who reported the absence of dendritic crystals or microsclerotia in the tested *A. radicina* carrot and parsley isolates on APDA, SNA, and V8 media.

**Table 1:** Visually determined colony, and growth characteristics of 10-day-old *A. radicina* isolates.

Medium	Colony color	Colony structure	Colony margins	Spores × 10^4^ mL^−1^	Conidiophore
Isolate Ar8					
V8	Black	Fluffy	Smooth	25.00	1 erected, far apart
SNA	Brown olive-green	Inside the media	Irregular	18.75	1 erected, far apart
APDA	Brown olive-green	Felted	Smooth	6.25	1-2 erected, far apart
Isolate Ar77					
V8	Black	Fluffy	Smooth	60.44	1 erected, far apart
SNA	Brown olive-green	Inside the media	Irregular	56.25	1 erected, far apart
APDA	Brown olive-green	Felted	Smooth	20.81	1-2 erected, far apart
Isolate Ar10					
V8	Black	Fluffy	Smooth	62.5	1-2 erected, far apart
SNA	Brown olive-green	Inside the media	Irregular	20.81	1-2 erected, far apart
APDA	Brown olive-green	Felted	Smooth	20.44	1-2 erected, far apart

The fungus produces 3.0–6.5 μm wide septate hyphae with obvious constrictions on the septa, generating a greyish-black or bluish-black mycelium. Conidiophores were simple, erect with 6–8 transverse septa, bearing solitary conidia, which are 105.4–182.9 μm long × 6.0–6.3 μm wide (av. 138.73 × 6.2 μm), unbranched, straight, septate, flexuous, and colored pale to olivaceous brown. The conidia were yellow to brown in color, non-catenulate, multicellular, beakless conidia measuring 18.6–52.7 × 12.4–27.9 (average; 36.57 × 21.88 μm) width, having 3–8 transverse and 1–3 longitudinal septa. Conidia with 1–3 short-tapered beaks 38.44–67.27 μm (average; 51.24 μm) were observed especially in the fully mature ones (Fig. 5).

These results are in line with those of Ellis and Holliday [52], who stated slight differences in measurements i.e., 3–7 transverse septa with 9–27 (*av*. 19) μm width and ≥1 longitudinal septum, with 27–57 (*av*. 38) μm length. Also, Saude and Hausbeck [57] stated dimensions of 35–45 × 15–18 μm of the mature conidia, with 3–8 transverse and 1–4 longitudinal septa. Similarly, the morphology of conidia of three *A. radicina* isolates corresponded to those reported by Kathe et al. [56] who recorded measurements of 2–5 transverse septa, with a length of 30–52 μm and a width of 19–24 μm. Based on morphology, pathogens were identified as *A. radicina* [30,31].

### 3.3 Pathogenicity test

Ten days after artificial infection, the typical symptoms of those detected in the fields appeared on all infected plants, in comparison to the symptomless of the control plants. Elongated brown lesions were observed on leaves with varying severities, covering 21–86 % of the leaf necrotic area. In general, the disease reduced plant health. *A. radicina* was reisolated again from 100 % of the infected leaves (n = 50), achieving Koch's postulates. Thus, the cause of coriander leaf blight was confirmed as *A. radicina*. To our knowledge, no previous work reports *A. radicina* as causative of foliar disease on coriander.

*Alternaria* spp. was previously reported as a causative of leaf blight with various severity degrees. *Alternaria petroselini*, *A. dauci,* and *A. radicina* were comparatively more violent than *A. alternata* [16]. *Alternaria petroselini* [58] and *A. alternata* [59] as well as *A. dauci* [11] were proven as pathogens associated with fennel and coriander plants, respectively. Herein, *A. radicina* was reported as a new pathogen on coriander plants for the first time ever.

### 3.4 Molecular identification

#### 3.4.1 ITS ribosomal RNA

**Fig. 6:**
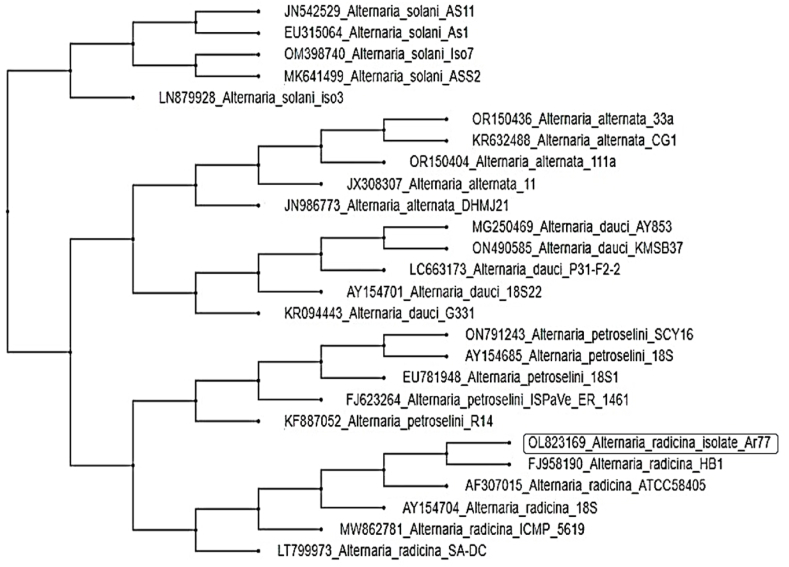
The constructed phylogeny using sequence analysis of the ITS gene of *Alternaria radicina* Ar77 (OL823169, highlighted in a black box) and similar sequences of *Alternaria* spp., as observed in the GenBank. The tree was constructed utilizing the bootstrap method with 1000 replications.

*A. radicina* Ar77 was additionally verified through molecular identification using ITS ribosomal RNA. The sequence of 18S rRNA gene was assessed and the phylogenetic tree was constructed (Fig. 6). The tree comprised two main groups, the first group contained *A. solani*. While the second main group is subdivided into *A. alternata* and *A. dauci*, clustered in the first subgroup. It was found that the Ar77 strain exhibited a high similarity and was located with the *A. radicina* formerly identified on GenBank. The morphological identification is compatible with the current molecular one. The GenBank accession number of *A. radicina* Ar77 was received as OL823169.

**Fig. 7:**
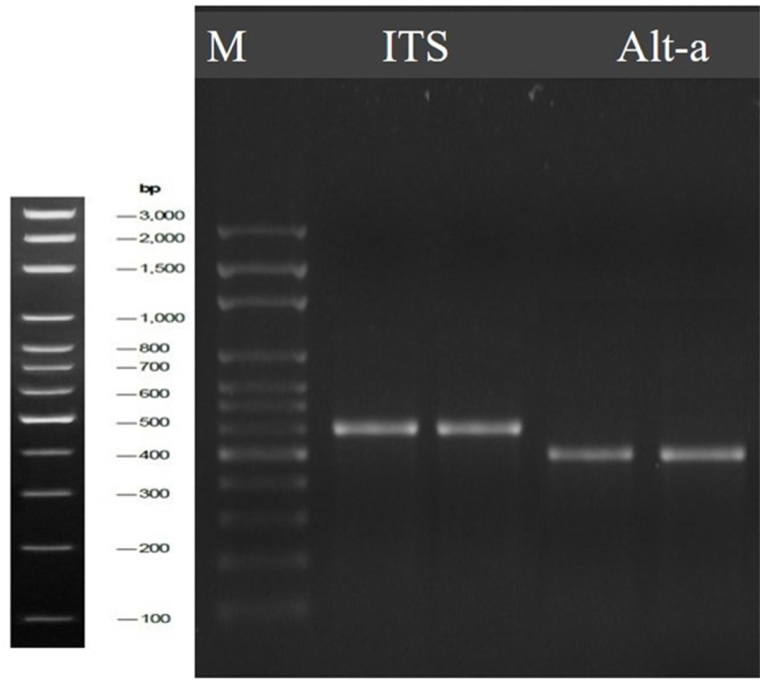
Agarose gel electrophoresis of the PCR bands of the product of the amplified ITS, and *Alt-a-1* fragment of the fungal strain.

Molecular identification is usually performed to ensure accuracy and confirmation of the morphological one. The ITS technique is a faultless means for molecular identification, representing a specific and highly sensitive for the rapid and accurate identification of numerous fungi. The phylogeny that was created from the fully annotated and well-identified sequence shows a strong association with the other similar fungi in the GenBank. The ITS region's nucleotide sequencing is amplified using the ITS primers, and the agarose gel electrophoresis is presented in Fig. (7), and Fig. S1. Several fungal groups share this fragment region's uniformity. Thus, the interspecific and, occasionally, intraspecific variation among species can be revealed [60].

This region can be adequate for identifying fungi at the species level since the sequences within this non-functional region often exhibit significant variability among fungal species [61]. Technically, the ITS sections can be easily amplified from a small sample of DNA due to the repetitive or multi-copy nature of the rDNA [28]. Therefore, compared to most other markers, sequencing the nucleotide of the ITS region is thought to yield speedy and extremely exact identification results. Additionally, a huge variety of fungi can be identified using this technique [62]. Interestingly, the molecular identification of *A. radicina* Ar77 (OL823169) came in agreement with the morphological characterization and confirmed the classification position of the pathogenic fungus. Thus, *Alternaria radicina* falls within the taxonomic classification of Eukaryota, specifically belonging to the Kingdom of Fungi. It is categorized under the Phylum: Ascomycota, Class: Loculoascomycetes, Order: Pleosporales, and Family: Pleosporaceae. Further, its genus is identified as *Alternaria*, with the species designation being *radicina*. This systematic classification provides a comprehensive understanding of the organism's phylogenetic placement within the fungal kingdom [63].

#### 3.4.2 Analysis of *Alt-a-1* gene of *A. radicina* Ar77

**Fig. 8:**
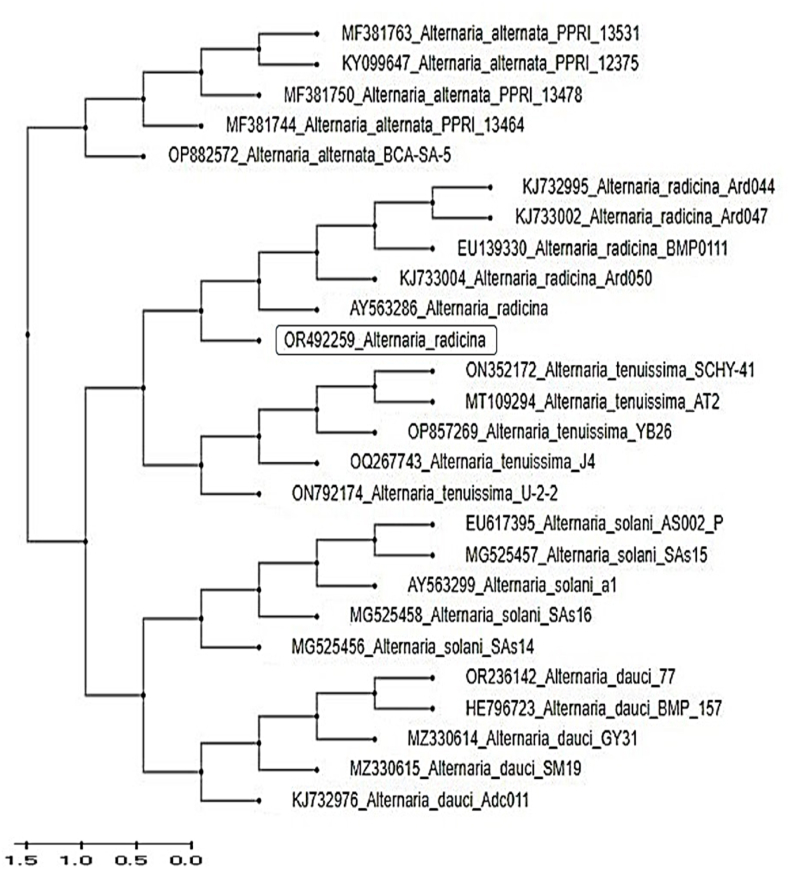
A phylogenetic tree generated through sequence analysis of the *Alt-a-1* gene, illustrating the positioning of the *A. radicina* Ar77 gene (GenBank number: OR492259, highlighted in a black box) amidst comparable sequences of *Alternaria* spp. sourced from the GenBank database. The bootstrap method with 1000 replications was used, and an external group was employed for reference.

The sequence of *A. radicina* (accession number OR492259) was analyzed. The results of the BLAST matching and phylogenetic analysis (Fig. 8) reveal the genetic relationships and evolutionary connections of the studied gene. BLAST matching allows for the identification of similar sequences in existing databases, shedding light on the genetic similarity with other known species.

The agarose gel electrophoresis of the *Alt-a-1* gene is presented in Fig. (7), and Fig. S1, and the newly generated Alt-gene sequence was aligned against other similar GenBank accessions, with a query coverage of 98–99 % (Table 2). This resulted in 500bp sequences of the Alt regions of *Alternaria*. Sequence alignment analysis revealed that the *Alt* gene sequences of *A. radicina* are 99.8 % identical in length. The phylogenetic tree of the *Alt* gene sequences of *A. radicina* was constructed using the sequences stored in the GenBank database. The phylogenetic tree comprised two main branches, the first main group contained *A. radicina*, while the second main group contained *A. solani, A. dauci A. tenuissima,* and *A. alternaria* with formerly identified representative *Alternaria* spp. This confirms the preceding morphological and molecular identifications.

**Table 2:** Retrieved *Alt-a-1* gene sequences from the GenBank database for related *Alternaria* spp. with a similarity percentage exceeding 98.55 %.

Fungus	Accession number	Query cove, %	E-value	Similarity, %
*Alternaria radicina*	KJ733002.1	99	0	99.78
*Alternaria radicina*	KJ733004.1	99	0	99.78
*Alternaria radicina*	EU139330.1	99	0	99.78
*Alternaria radicina*	AY563286.1	99	0	99.78
*Alternaria radicina*	KJ732995.1	99	0	99.78
*Alternaria radicina*	KJ733011.1	99	0	99.78
*Alternaria radicina*	KJ732991.1	99	0	99.74
*Alternaria radicina*	EU139346.1	98	0	98.68
*Alternaria alternaria*	MF381763.1	98	0	98.55
*Alternaria alternaria*	OP882572.1	98	0	98.55

The fungus was characterized by conducting a phylogenetic analysis of the DNA sequence within the *Alt-a-1* gene region [64]. To enable rapid DNA detection of various *Alternaria* spp., several authors [[65], [66], [67]] employed a PCR that utilized oligonucleotide primers targeting the *Alt-a-1* gene, which was discovered to be predominant in *Alternaria* spp., making it both challenging and valuable for identifying *Alternaria* spp. *Alt-a-1* serves as a key allergen produced by *A. alternata* and is frequently employed in allergy testing for identification and diagnosis. This protein is present in mold spores and aids in diagnosing allergies specific to *Alternaria* spp. [39]. However, it is important to note that the *Alt-a-1* gene alone may not be sufficient for accurately identifying *Alternaria* spp. For this reason, it is advisable to combine the ITS technique with gene identification to achieve precise identification [68,69]. This gene, in addition to morphological characteristics and ITS regions, can be utilized for species identification [70,71].

Accordingly, molecular studies confirm the pathogen identification as *A. radiciana*. In general, the study employed a multi-pronged approach, including cultural, macroscopic, and microscopic characteristics, subsequently, molecular techniques were utilized to corroborate these findings. This combination of methods provides a robust foundation for the identification of *A. radiciana* pathogen.

### 3.5 Enzymatic profile of *A. radicina* Ar77

**Fig. 9:**
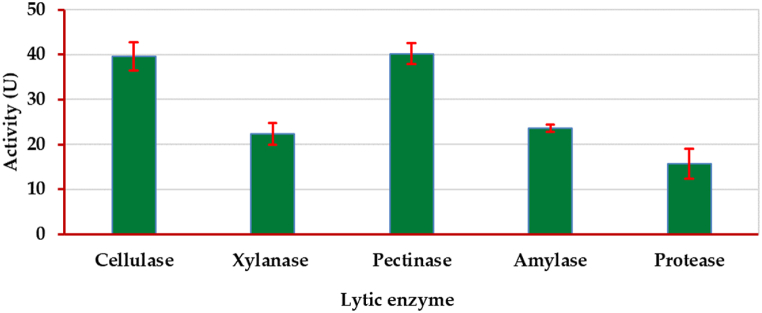
The profile of the lytic activity of *Alternaria radicina* Ar77 on coriander plant tissue (*n* = 3, ±SD).

To determine the pattern and mechanism of action of the pathogenic fungus under test's ability to hydrolyze plant tissues, the enzymatic system of *A. radicina* Ar77 was investigated (Fig. 9). The action of numerous hydrolytic enzymes i.e., cellulase xylanase, pectinase, amylase, and proteinase, was evident by 39.67, 22.34, 40.17, 23.59, and 15.67 U, respectively.

The study aimed to elucidate the invasive potential of the recently identified fungal pathogen, Ar77, on coriander plant tissue. The enzyme analysis was conducted to substantiate the pathogenicity of the isolated fungus, as these hydrolytic enzymes have a pivotal job in the maceration of tissues at the injection site, marking the initial phase of the microbial invasion. The results revealed the presence of various lytic enzymes. The pathogenicity of this phytopathogenic fungus was discovered to be closely associated with its lytic activity, underscoring the significance of these enzymes in the progression of the disease [29,45]. Therefore, coriander plant tissue was used to test the current pathogenic fungal isolate's lytic activity. Lytic enzymes are essential for the pathogenesis process because they help degrade the barriers presented in complex plant structures, preventing phytopathogen invasion. Consequently, discernible distinctions in enzymatic profiles between virulent and avirulent bacteria have been established [[71], [72], [73]]. Therefore, the study assayed cellulase, xylanase, pectinase, amylase, and protease activities in the present pathogenic fungus. These cell wall-degrading enzymes collectively contribute to the pathogenicity process, facilitating the fungal penetration of plant tissue [28,40].

Cellulase converts cellulose into a single unit of glucose tissue [29,40,45]. Significant xylanase activity was observed, indicating that this fungus could catalyze the breakdown of xylan found in plant tissue's hemicellulose [27,45]. Pectinase breaks down pectin by cleaving the 1,4-glycosidic link, releasing monomers of galacturonic acid [29,42]. Amylase breaks down starch, releasing glucose units, while proteases hydrolyze the nitrogenous portion of the tissue, releasing peptides and amino acids [28,73,74].

It is possible that the activity of the enzymatic consortium results in the degradation of tissues and facilitates the pathogen's infection, which is a crucial process for pathogenicity. Infection of plants is additionally threatened by lytic enzymes. In conclusion, the current fungus can generate a significant infection and its complementary profile of lytic enzymes may play a vital role in this respect.

### 3.6 Seed-borne transmission

Seeds are crucial for crop propagation but also serve as primary vectors for transmitting pathogens. Seed-borne pathogens (bacteria, fungi, viruses, and nematodes) can infect plants at all stages, causing diseases, reducing yield, and compromising seed quality. These pathogens can reside within or on seeds or be associated with contaminants. Infected seeds transmit pathogens to subsequent crops, potentially leading to widespread disease outbreaks and mycotoxin contamination, posing risks to both humans and animals. To monitor the spread of fungal pathogens across generations and their impact on seed health, seed health tests are essential [[75], [76], [77]].

**Fig. 10:**
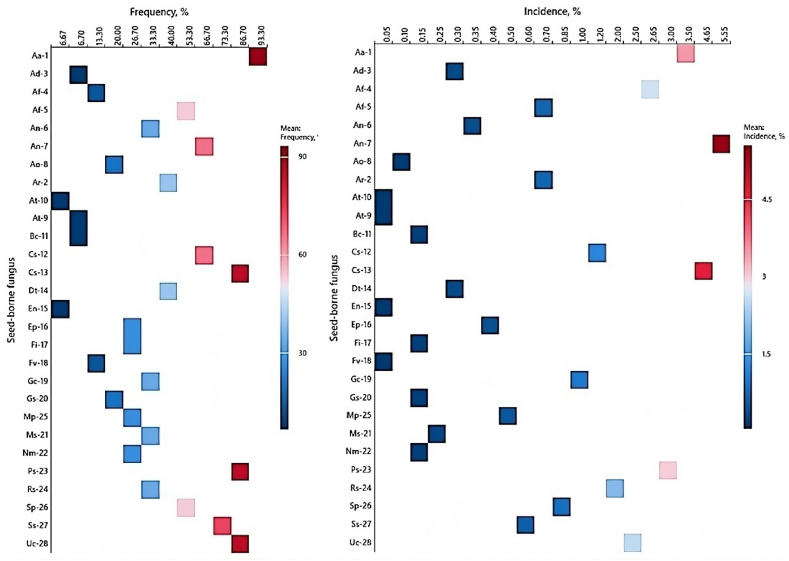
Heatmap of the occurrence of coriander seed-borne fungi, showing the fungal frequencies and incidences (% infected seeds) of the 28 fungi associated with coriander seeds. Gradients of the frequency bar on the right vary from low (blue) to high (red). Aa-1 = *Alternaria alternata*, Ar-2 = *Alternaria radicina*, Ad-3 = *Alternaria dauci*, Af-4 = *Aspergillus flavus*, Af-5 = *Aspergillus fumigatus*, An-6 = *Aspergillus nidulans*, An-7 = *Aspergillus niger*, Ao-8 = *Aspergillus ochraceus*, At-9 = *Aspergillus tamari*, At-10 = *Aspergillus terreus*, Bc-11 = *Botrytis cinerea*, Cs-12 = *Chaetomium* spp., Cs-13 = *Cladosporium* spp., Dt-14 = *Drechslera tetramera*, En-15 = *Emericella nidulans*, Ep-16 = *Epicoccum purpurascens*, Fi-17 = *Fusarium incarnatum*, Fv-18 = *Fusarium verticillioides*, Gc-19 = *Geotrichum candidum*, Gs-20 = *Gliocladium* spp., Ms-21 = *Mucor* spp., Nm-22 = *Nigrospora* spp., Ps-23 = *Penicillium* spp., Rs-24 = *Rhizopus stolonifer*, Mp-25 = *Macrophomina phaseolina*, Sp-26 = *Stachybotrys* spp., Ss-27 = *Stemphylium* spp., Uc-28 = *Ulocladium chartarum*.

In this respect, various coriander seeds were surveyed for seed-borne mycoflora for elucidation of the possible distribution of the new coriander pathogen; *Alternaria rdicina* Ar77. The heatmap (Fig. 10, and Table S1) was used to evaluate the frequency, and incidence of each of the seed-borne fungus on coriander seeds.

Upon performing the SMB procedure, 28 fungal species, comprising 19 genera, were detected from coriander seeds. Among them, *Alternaria alternata*, *Cladosporium* spp., *Penicillium* spp., *Stemphylium* spp., and *Ulocladium chartarum* had the maximum average frequency (>73 %). *Alternaria radicina*, *Aspergillus fumigatus, A. niger, Chaetomium* sp.*, Drechslera tetramera,* and *Stachybotrys* spp. came next with a frequency of ≥40 %) and their incidence (0.7 ± 0.3) ranged from 0.50 to 5.55 %. *F. incarnatum* and *Macrophomina phaseolina* recorded a lower frequency (26.7 % for both), followed by *Fusarium verticillioides* (13.3 %), while *Alternaria dauci* and *Botrytis cineara* were less frequently recorded (6.70, for both). In this respect, highly infected seed samples especially by *A. radicina* pathogen were usually characterized by discolored, smaller and shriveled symptoms, with color ranging from light brown to dark brown or even black (Fig. 3).

Our findings align with several prior studies, confirming the infection and transmission of *A. radicina* pathogen by the seed of other investigated *Apiaceae* host plants worldwide. There have been multiple cases of *A. radicina* infection in carrot seeds, with levels averaging 35 % [15], 37.5–63.5 % [78], and up to 10 % [16]. The pathogen was also detected on anise seeds with a frequency percentage that reached 54 % and a mean intensity percentage of 2.88–6.22 % [23] as well as parsley seeds with a high percentage [79]. The fungus is known primarily as a seed-borne pathogen of other *Apiaceae* plants i.e., celery and celeriac, caraway, dill, and fennel, and occasionally found in the seed of parsnip causing foliar blight and stalk/root rot disease [13,[17], [18], [19],^52^].

In this respect, Kim and Mathur [78] reported that *A. radicina* hyphae were in the inner pericarp layers and seedcoat, and endosperm of the carrot seeds but not in the embryo. *A. radicina* is characterized by the high yellow pigment level on APDA due to radicinin biosynthesis [53]. *A. radicina* can also create minor amounts of the hazardous metabolites radicinol and epiradicinol [[80], [81]]. Radicinin is relevant to *A. radicina*'s pathogenicity on carrots and has been discovered on naturally infested carrots [80]. In this connection, Farrar et al. [13] described the beginning black rot disease cycle by the planting of *A. radicina*–infested carrot seed, where the pathogen is transmitted from them during germination to infect the hypocotyl, resulting in black necrosis and preemergence damping-off. Seedling mortality due to infection occurs at or near the soil line. The pathogen then sporulates, and spores are disseminated via wind, rain, or irrigation water to other seedlings, leading to lowered stands and yields. The pathogen is also transmitted through the soil, where it can survive in plant remainder or as microsclerotia or spores, facilitating disease transmission [13,15,17,19,75].

## 4 Conclusion

In this study, the *A*. *radicina* pathogen causing a new leaf blight in coriander plants is detected and identified for the first time in Egypt in 2021. The disease negatively impacted plant health and seed quality. Pathogenicity testing, morphological and molecular identifications (ITS and specific *Alt-a-1* gene), and Koch's postulates confirmed *A. radicina* as the causative agent. The pathogen showed lytic activity that plays a role in pathogenicity development. Additionally, the study found that seeds played a central role in transmitting the pathogen, as *A. radicina* was isolated from coriander seeds along with other fungal pathogens and saprophytes. According to the results of this study, it is recommended to implement seed treatment measures to prevent the transmission of *A. radicina* during agricultural seasons. Treating seeds before planting can help reduce the incidence and spread of the disease, thus improving coriander plant health and seed quality. Seed-health test trials should be conducted regularly on coriander to monitor the presence of the new *A. radicina* pathogen on. Future research could focus on developing more targeted and effective seed treatment methods to combat *Alternaria* leaf spot disease affecting coriander.

## CRediT authorship contribution statement

**Khalid M. Ghoneem:** Writing – review & editing, Writing – original draft, Visualization, Data curation, Conceptualization. **Ehsan M. Rashad:** Writing – original draft, Methodology, Investigation, Data curation, Conceptualization. **Abdulaziz A. Al-Askar:** Writing – original draft, Resources, Project administration, Funding acquisition. **Yosra A. Helmy:** Writing – review & editing, Writing – original draft, Methodology, Investigation. **Seham M.A. El-Gamal:** Writing – review & editing, Writing – original draft, Methodology, Investigation, Formal analysis, Data curation, Conceptualization. **Shafik D. Ibrahim:** Writing – original draft, Validation, Formal analysis, Data curation. **WesamEldin I.A. Saber:** Writing – review & editing, Writing – original draft, Project administration, Methodology, Formal analysis, Conceptualization.

## Data availability statement

All data generated or analyzed during this study are included in this published manuscript and Supplementary file. The sequences of the ITS gene of *Alternaria radicina* Ar77 (OL823169), and the *Alt-a-1* gene (OR492259) were deposited in the GenBank; https://www.ncbi.nlm.nih.gov/.

## Funding

This work is supported by Researchers Supporting Project number (RSP2024R505), 10.13039/501100002383King Saud University, Riyadh, Saudi Arabia.

## Declaration of competing interest

The authors declare that they have no known competing financial interests or personal relationships that could have appeared to influence the work reported in this paper.
