# Supplementary material for: Alternaria radicina; unveiling the cause, spread, and molecular basis of a novel coriander leaf blight disease in Egypt
Source: Heliyon. 2024 Dec 10;11(1):e41081. doi: 10.1016/j.heliyon.2024.e41081 (PMC11719364; doi:10.1016/j.heliyon.2024.e41081)
Supplement: Multimedia component 1 [file mmc1.docx]

*Alternaria radicina*; unveiling the cause, spread, and molecular basis of a novel coriander leaf blight disease in Egypt

Khalid M. Ghoneem ^a,^* , Ehsan M. Rashad ^a^ , Abdulaziz A. Al-Askar ^b^ , Yosra A. Helmy ^c^ , Seham M. A. El-Gamal ^d^, Shafik D. Ibrahim ^e^ and WesamEldin I. A. Saber ^f,^*

^a^ Department of Seed Pathology Research, Plant Pathology Research Institute, Agricultural Research Center, (ARC), Giza 12619, Egypt; [khalidghoneem@arc.sci.eg](mailto:khalidghoneem@arc.sci.eg) (KMG); [ehsanrashad78@arc.sci.eg](mailto:ehsanrashad78@arc.sci.eg) (EMR)

^b^ Department of Botany and Microbiology, Faculty of Science, King Saud University, Riyadh 11451, Saudi Arabia; [aalaskara@ksu.edu.sa](mailto:aalaskara@ksu.edu.sa)

^c^ Department of Veterinary Science, Martin-Gatton College of Agriculture, Food, and Environment, University of Kentucky, Lexington, KY 40546, USA; [yosra.helmy@uky.edu](mailto:yosra.helmy@uky.edu)

^d^ Department of Medicinal and Aromatic Plants Research, Horticulture Research Institute, Agricultural Research Center (ARC), Giza 12619, Egypt; [s_elgamal99@yahoo.com](mailto:s_elgamal99@yahoo.com)

^e^ Department of Genome Mapping, Agricultural Genetic Engineering Research Institute (AGERI), Agricultural Research Center (ARC), Giza, Egypt; [shafikdarwish2014@gmail.com](mailto:shafikdarwish2014@gmail.com)

^f^ Microbial Activity Unit, Department of Microbiology, Soils, Water and Environment Research Institute, Agricultural Research Center (ARC), Giza 12619, Egypt; E-mail: [wesameldin.saber@arc.sci.eg](mailto:wesameldin.saber@arc.sci.eg);

***** Correspondence: [khalidghoneem@arc.sci.eg](mailto:khalidghoneem@arc.sci.eg) (KMG); [wesameldin.saber@arc.sci.eg](mailto:wesameldin.saber@arc.sci.eg); [wiasaber@gmail.com](mailto:wiasaber@gmail.com) (WIAS)

**Section 1. Detection of lytic enzymes**

**Lytic enzyme activities (**cellulase**,** xylanase, polygalacturonase, α-amylase, and protease) were determined according to previously published methods. Briefly, cellulase activity was assessed (Saber et al. 2010) by measuring reducing sugars released from microcrystalline cellulose (0.5%, suspended in 0.05 M citrate buffer, pH 4.8) after incubation with the enzyme filtrate for 1 hour at 50 °C, using the dinitrosalicylic acid (DNS) method. Xylanase activity was determined similarly, employing 0.5% xylan as the substrate in 0.05 M citrate buffer (pH 4.8) and incubating for 30 minutes at 50 °C (Bailey et al. 2992). Polygalacturonase activity was measured by monitoring the release of reducing sugars from 0.5% pectin dissolved in 0.1 M sodium acetate buffer (pH 5.2) after incubation with the enzyme filtrate for 30 minutes at 40 °C (Bai et al. 2004). α-Amylase activity was determined by measuring the release of reducing sugars from 0.5% soluble starch dissolved in phosphate buffer (pH 6.5) following incubation with the enzyme filtrate for 10 minutes at 30 °C [Kathiresan, and Manivannan, 2006]. Reducing sugars released by the previous 4 enzymes were quantified using the DNS (Miller, 1959), calibrated using standard curves, and expressed as enzyme units (U), which are defined as the amount of enzyme releasing 1 µmol of glucose (cellulase and amylase), xylose (xylanase), or galacturonic acid (polygalacturonase) per gram biomass/min under the assay conditions for each enzyme.

Protease activity was assessed by incubating a casein-enzyme filtrate mixture for 10 minutes at 37 °C, followed by trichloroacetic acid precipitation. Released amino acids were quantified by measuring absorbance at 280 nm (Al-Askar, et al. 2022). Protease activity U was expressed as the release of one µg of tyrosine/g/min under test settings.

**Section 2. Occurrence of seed-borne fungi**

**Table S1.** Occurrence (frequencies and incidences) of coriander seed-borne fungi of the 28 fungi associated with coriander seeds.

| Seed-borne fungus | | Occurrence of seed-borne fungus | |
| --- | --- | --- | --- |
| Scientific name | Code | Frequency, % | Incidence, % |
| *A.alternata* | Aa-1 | 93.30 | 3.50 |
| *Alternaria radicina* | Ar-2 | 40.00 | 0.70 |
| *Alternaria dauci* | Ad-3 | 6.70 | 0.30 |
| *A. flavus* | Af-4 | 13.30 | 2.65 |
| *A. fumigatus* | Af-5 | 53.30 | 0.70 |
| *A. nidulans* | An-6 | 33.30 | 0.35 |
| *A.niger* | An-7 | 66.70 | 5.55 |
| *A. ochraceus* | Ao-8 | 20.00 | 0.10 |
| *A. tamarii* | At-9 | 6.70 | 0.05 |
| *A. terreus* | At-10 | 6.67 | 0.05 |
| *Botrytis cinerea* | Bc-11 | 6.70 | 0.15 |
| *Chaetomium spp.* | Cs-12 | 66.70 | 1.20 |
| *Cladosporium spp.* | Cs-13 | 86.70 | 4.65 |
| *Drechslera tetramera* | Dt-14 | 40.00 | 0.30 |
| *Emericella nidulans* | En-15 | 6.67 | 0.05 |
| *Epicoccum purpurascens* | Ep-16 | 26.70 | 0.40 |
| *Fusarium incarnatum* | Fi-17 | 26.70 | 0.15 |
| *F. verticillioides* | Fv-18 | 13.30 | 0.05 |
| *Geotrichum candidum* | Gc-19 | 33.30 | 1.00 |
| *Gliocladium* spp*.* | Gs-20 | 20.00 | 0.15 |
| *Mucor* spp*.* | Ms-21 | 33.30 | 0.25 |
| *Nigrospora* spp*.* | Nm-22 | 26.70 | 0.15 |
| *Penicillium* spp*.* | Ps-23 | 86.70 | 3.00 |
| *Rhizopus* *stolonifer* | Rs-24 | 33.30 | 2.00 |
| *Macrophomina phaseolina* | Mp-25 | 26.70 | 0.50 |
| *Stachybotrys* spp*.* | Sp-26 | 53.30 | 0.85 |
| *Stmphylium* spp*.* | Ss-27 | 73.30 | 0.60 |
| *Ulocladium chartarum* | Uc-28 | 86.70 | 2.50 |


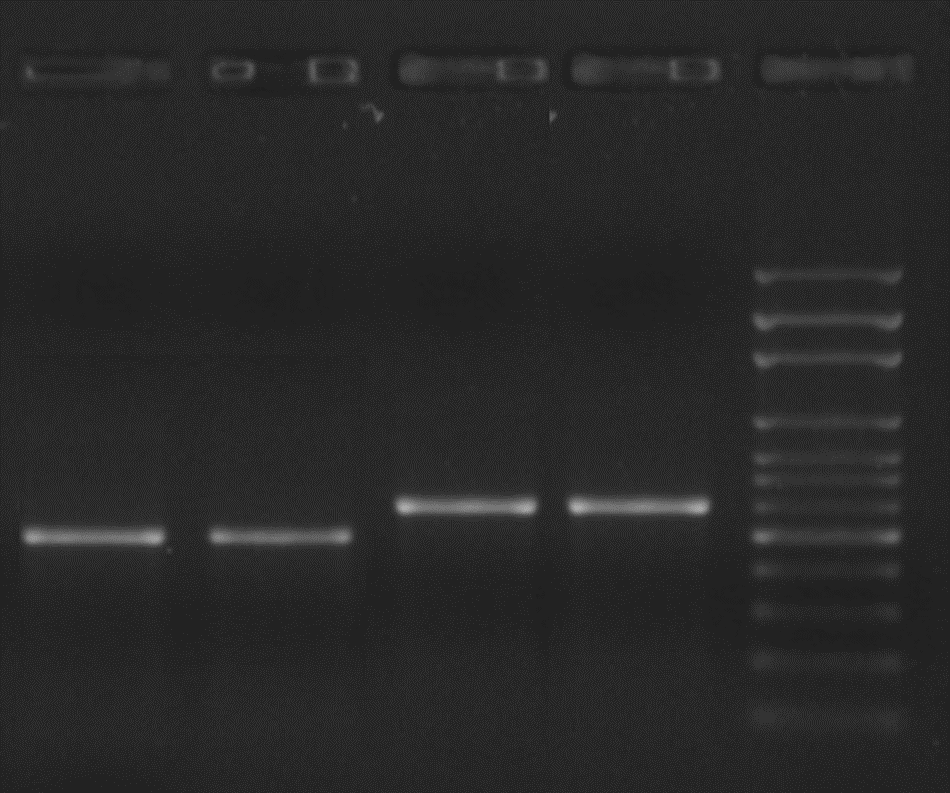


Fig. S1. Agarose gel electrophoresis of the PCR bands of the amplified ITS, and *Alt-a-1* fragment.

**References**

Saber, W.I.A., N.E. El-Naggar, S.A. AbdAl-Aziz, Bioconversion of lignocellulosic wastes into organic acids by cellulolytic rock phosphate-solubilizing fungal isolates grown under solid-state fermentation conditions, Research Journal of Microbiology 5 (2010) 1–20, https://doi.org/10.3923/jm.2010.1.20.

Bailey, M.J., P. Beily, K. Poutanen, Interlaboratory testing and methods for assay of xylanase activity, Journal of Biotechnology, 23 (1992) 257-270, https://doi.org/10.1016/0168-1656(92)90074-J.

Bai, Z.H., H.X. Zhang, H.Y. Qi, X.W. Peng, B.J. Li, Pectinase production by *Aspergillus niger* using wastewater in solid state fermentation for eliciting plant disease resistance, Bioresource Technology 95 (2004) 49–52, https://doi.org/10.1016/j.biortech.2003.06.006.

Kathiresan, K., S. Manivannan, Amylase production by *Penicillium fellutanum* isolated from mangrove rhizosphere soil, African Journal Biotechnology 5 (2006) 829–832, https://doi.org/10.3923/jm.2006.438.442.

Miller, G.L., Use of dinitrosalicylic acid reagent for determination of reducing sugars, Analytical Chemistry 31 (1959) 426–428, https://doi.org/10.1021/ac60147a030.

Al-Askar, A.A., E.M. Rashad, Z. Moussa, K.M. Ghoneem, A.A. Mostafa, F.O. Al-Otibi, W.I.A. Saber, A novel endophytic *Trichoderma longibrachiatum* WKA55 with biologically active metabolites for promoting germination and reducing mycotoxinogenic fungi of peanut, Frontiers in Microbiology 13 (2022) 772417, https://doi.org/10.3389/fmicb.2022.772417.
